# The Epiphytic Genus *Gambierdiscus* (Dinophyceae) in the Kermadec Islands and Zealandia Regions of the Southwestern Pacific and the Associated Risk of Ciguatera Fish Poisoning

**DOI:** 10.3390/md15070219

**Published:** 2017-07-11

**Authors:** Lesley L. Rhodes, Kirsty F. Smith, Sam Murray, D. Tim Harwood, Tom Trnski, Rex Munday

**Affiliations:** 1 Cawthron Institute, Private Bag 2, Nelson 7042, New Zealand; kirsty.smith@cawthron.org.nz (K.F.S.); Sam.murray@cawthron.org.nz (S.M.); tim.harwood@cawthron.org.nz (D.T.H.); 2 Auckland War Memorial Museum, Private Bag 92018, Victoria Street West, Auckland 1010, New Zealand; ttrnski@aucklandmuseum.com; 3 AgResearch, Ruakura Research Centre, 10 Bisley Road, Private Bag 3240, Hamilton 3214, New Zealand; rex.munday@agresearch.co.nz

**Keywords:** *Gambierdiscus*, ciguatera fish poisoning, ciguatoxins, maitotoxin, Kermadec Islands, New Zealand

## Abstract

Species in the genus *Gambierdiscus* produce ciguatoxins (CTXs) and/or maitotoxins (MTXs), which may cause ciguatera fish poisoning (CFP) in humans if contaminated fish are consumed. Species of *Gambierdiscus* have previously been isolated from macroalgae at Rangitahua (Raoul Island and North Meyer Islands, northern Kermadec Islands), and the opportunity was taken to sample for *Gambierdiscus* at the more southerly Macauley Island during an expedition in 2016. *Gambierdiscus* cells were isolated, cultured, and DNA extracted and sequenced to determine the species present. Bulk cultures were tested for CTXs and MTXs by liquid chromatography-mass spectrometry (LC-MS/MS). The species isolated were *G. australes*, which produced MTX-1 (ranging from 3 to 36 pg/cell), and *G. polynesiensis*, which produced neither MTX-1 nor, unusually, any known CTXs. Isolates of both species produced putative MTX-3. The risk of fish, particularly herbivorous fish, causing CFP in the Zealandia and Kermadec Islands region is real, although in mainland New Zealand the risk is currently low. Both *Gambierdiscus* and *Fukuyoa* have been recorded in the sub-tropical northern region of New Zealand, and so the risk may increase with warming seas and shift in the distribution of *Gambierdiscus* species.

## 1. Introduction

*Gambierdiscus* Adachi and Fukuyo is an epiphytic dinoflagellate genus found attached to macroalgae, dead corals and volcanic sands throughout the world’s tropical regions [1,2]. In the Pacific region, *Gambierdiscus* is found on macroalgae, particularly filamentous red macroalgae, coralline turfs, and the calcareous green genus, *Halimeda*, and on the volcanic debris that is common in the active zones. 

Species in the *Gambierdiscus* genus are the causative organisms of ciguatera fish poisoning (CFP) [2]. The toxins produced by some *Gambierdiscus* species include ciguatoxins (CTXs), maitotoxin (MTX) and its analogues, gambieric acids, gambieroxide, gambierol and gambierone [3]. CTXs are considered the main cause of CFP, the economic impacts of which on Pacific Island communities are just beginning to be recognized. The illness is widely under-reported and may include gastrointestinal and neurological effects, and deaths have occurred [4,5]. Folk remedies based on the local floras are used in many Pacific Islands, and intravenous mannitol has been suggested as a possible treatment [6], although the efficacy of this substance has been disputed [7]. Currently, treatment is largely supportive and symptom-driven, as a proven antidote has not yet been developed for CFP [5]. 

**Figure 1:**
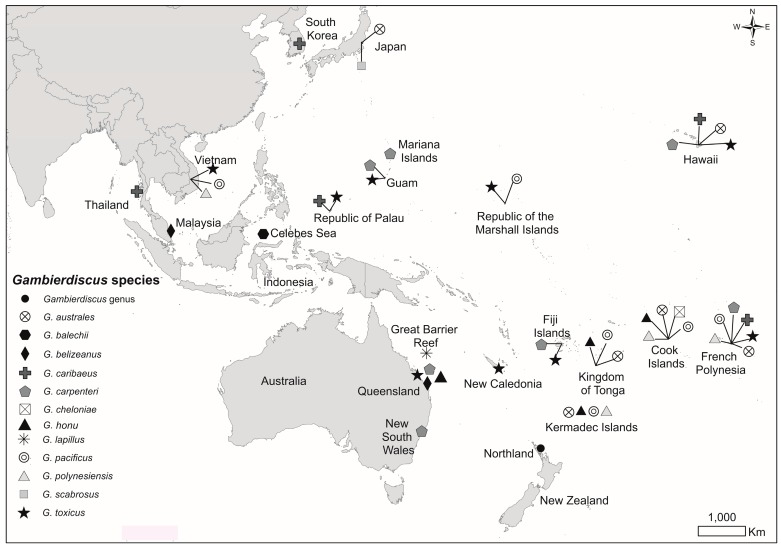
Map of the Pacific region showing the geographic distribution of *Gambierdiscus* species (as of April 2017; updated from Rhodes et al., 2017a,b [8,9]).

The number of new *Gambierdiscus* species being described annually has increased. At the beginning of 2017, fifteen species of *Gambierdiscus* had been described globally with three of these being described in 2016, an increase of nearly 30% in that one year [8]. One species, *G. honu*, described in 2017, was isolated from Rangitahua/North Meyer Island, Kermadec Islands [9]. Currently, twelve *Gambierdiscus* species of the fifteen described are known in the Pacific region, although the CTX producer, *G. polynesiensis*, has been isolated much less frequently from samples than the MTX-1 producer, *G. australes* [8] (Figure 1).

CFP was recently identified as a potential risk in the temperate southwest Pacific based on the emergence of *G. carpenteri* blooms in the temperate waters of New South Wales, Australia [10]. *G. carpenteri* has previously been found only in the tropical waters of northern Australia and, while *G. carpenteri* is not a CTX producer, other CTX producing species may also move into this region in time. The genus *Gambierdiscus* and the closely related genus *Fukuyoa* (previously transferred from the genus *Gambierdiscus*) have also been reported from New Zealand’s northern, mainland coastal waters [11,12] and the same potential risk scenario applies.

The expected effects of climate change include changing currents and warming seas [13,14]. The risk of CFP is expected to increase as *Gambierdiscus* is delivered to more temperate regions [10], where cells may become adapted to cooler conditions. Another likely scenario is that cells will be transported to previously temperate habitats which have become tropicalized [15]. A more haphazard way that the biogeographic occurrence of *Gambierdiscus* may expand, and therefore CFP risk occur in new areas, is via rafting pumice, following submerged volcanic activity [16]. For example, pumice from the Havre seamount eruption in the Kermadec Islands (2012) was tracked over thousands of kilometers of the Pacific Ocean [17]. These rafts are not uncommon in the southwestern Pacific and the pumice is often fouled with mixed populations of flora and fauna [17].

Mainland New Zealand is situated on what is now considered, albeit controversially, to be a submerged geological continent, Zealandia [18]. Zealandia reaches (and includes) New Caledonia to the northwest, where CFP has been well documented [19] and where *G. toxicus* has been reported [20,21]. There has been speculation that CTX-like compounds found in giant clams (*Tridacna* spp.) in New Caledonia were due to cyanobacteria [22], but this remains hypothetical. Clams may take up *Gambierdiscus* and accumulate CTX [23] and storage of toxins produced by dinoflagellates (for example, saxitoxins) in clam siphons for extended periods of time is well documented [24]. 

**Figure 2:**
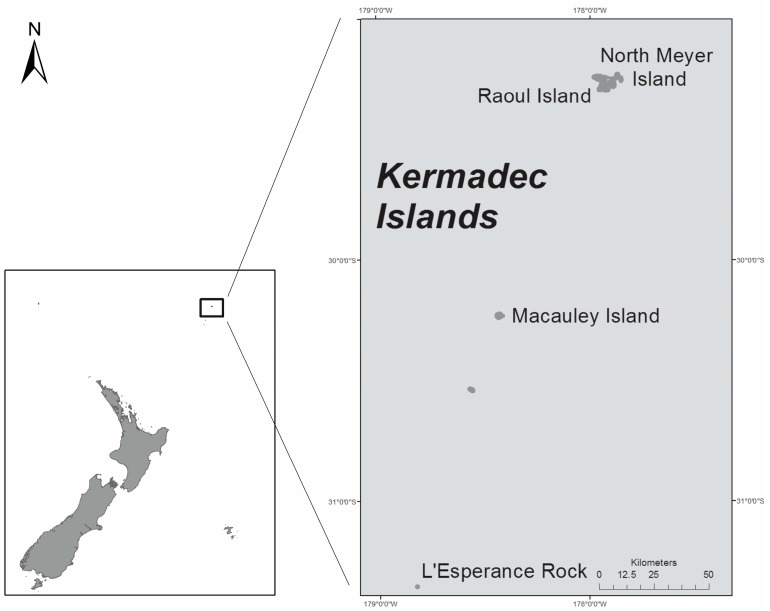
Map of New Zealand (**L**) and enlargement of Kermadec Islands (**R**) showing the sampling sites at Rangitahua (Raoul and North Meyer Islands), Macauley Island and L’Esperance Rock.

Rangitahua (Kermadec Islands) is a New Zealand territory, geographically distinct from Zealandia. They form a subtropical island arc in the South Pacific Ocean. The islands extend 800–1000 km northeast of New Zealand’s North Island, and a similar distance southwest of Tonga (Figure 2). The islands are currently uninhabited, except for the permanently-manned Raoul Island Station, the northernmost outpost of New Zealand. Rangitahua is considered a stepping stone for fish migrations from tropical islands in the region migrating to northern New Zealand and the risk of tropical fish arriving in New Zealand’s northern coastal waters contaminated with CTXs is real [25].

The first scientific expedition to the Kermadec Islands, on HMS *Herald*, was in 1854 [26], but it was not until 1984 that a dedicated marine survey was undertaken. The largest and most comprehensive marine survey was undertaken on board the RV *Braveheart* in 2011 [27]. Prior to 2011, the only micro-algae in the class Dinophyceae recorded from the Kermadec Islands were endosymbionts of corals in the genus *Symbiodinium* (Order Suessiales) [28]. During further expeditions in 2013 and 2015, epiphytic micro-algae were collected from macroalgae and coralline turfs and the dinoflagellate genera *Gambierdiscus, Amphidinium, Ostreopsis, Prorocentrum* and *Coolia* were recorded from Rangitahua [25,29,30]. In this study, dinoflagellates isolated from macroalgae on Macauley Island, October 2016, were identified and their toxin production, if any, determined.

## 2. Results

### 2.1. Dinoflagellate Species Identified

Sea water samples collected from mixed macroalgae at Macauley Island were transported to the Cawthron Institute where dinoflagellates were isolated and cultured. Successful cultures included species in the genera *Gambierdiscus, Ostreopsis* and *Coolia* and all were identified by analysis of DNA sequence data (Table 1).

**Figure 3:**
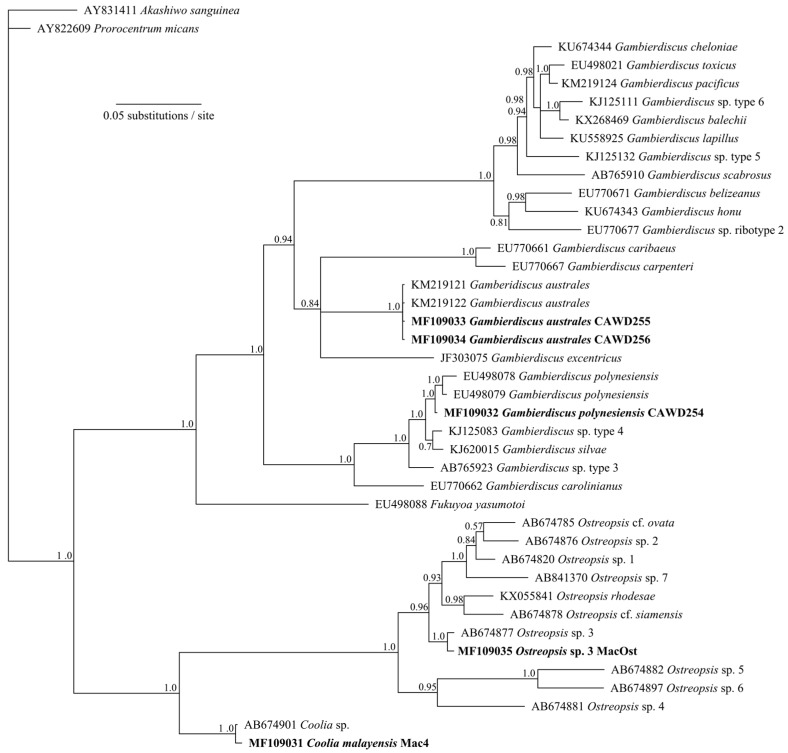
Phylogenetic analysis of large sub-unit (LSU D8–10 region) sequences obtained from dinoflagellate species isolated during this study (in bold) using Bayesian analyses. Values at nodes represent Bayesian posterior probability support. Scale bar is substitutions per site.

*Gambierdiscus australes* was the most frequently isolated species from the Macauley Island samples and twenty-seven isolates were cultured and analysed in order to select MTX-1 and MTX-3 producers for further research purposes, including production of reference materials. Two isolates of *G. polynesiensis* were also successfully cultured and one is maintained in the Cawthron Institute Culture Collection of Micro-algae (CICCM), a nationally significant collection, as CAWD254. The *Gambierdiscus* species were identified by analysis of large subunit ribosomal RNA sequences (D8–10 region; large sub-unit (LSU)) (Table 1; Figure 3).

Co-occurring species were *Ostreopsis* sp. 3 [31] and *C. malayensis* [32] (GenBank accession numbers MF109035 and MF109031 respectively). *Prorocentrum* and *Amphidinium* cells were identified by light microscopy, but were not isolated. Some samples contained high concentrations of foraminifera.

Samples were also collected from L’Esperance Rock, the southern Kermadec Islands, but no dinoflagellates were observed. These samples contained high concentrations of diatom frustules as well as nematodes and cyanobacteria (cf. *Oscillatoria* spp.).

### 2.2. Toxin Production

All the *G. australes* isolates tested produced MTX-1 and MTX-3. The range of concentrations of MTX-1 per cell was 3–36 pg/cell, with a mean of 17 pg/cell and median of 18 pg/cell (Table 1). Two of the best producers (Mac1-b and Mac3-c) were submitted to the Cawthron Institute Culture Collection of Micro-algae (CICCM), as CAWD255 and CAWD256 respectively, for on-going maintenance.

The *G. polynesiensis* isolate CAWD254 did not produce any known CTXs or MTX-1, but did produce MTX-3.

## 3. Discussion

In late October 2016, an expedition aboard the RV *Tangaroa* visited the Kermadec Islands, including Macauley Island (Figure 2), to undertake marine biodiversity surveys. Samples were collected from macroalgae for the isolation of *Gambierdiscus*. The study focused on Macauley Island, but included two samples from L’Esperance Rock. However, no dinoflagellates were found in the latter samples.

Isolates of *Gambierdiscus* from Macauley Island were predominantly the MTX-1 producer, *G. australes*, and the best producers have been retained for further research, including full characterisation of MTX-3. The range of MTX-1 concentrations per cell, as determined by liquid chromatography-mass spectrometry (LC-MS/MS) showed a wide variation. A great variation was also determined by Pisapia et al. (2017) using neuro-2a and erythrocyte lysis bioassays [33]. The toxicity of a selection of these isolates is summarized by Munday et al. (this issue of *Marine Drugs*) [34].

The known CTX producer, *G. polynesiensis* was also isolated from Macauley Island (CAWD254). The Macauley Island isolate did not, unusually, produce any known CTXs (or MTX-1), CTX being considered the main cause of CFP. The presence of this species suggests that the Kermadec Islands do offer suitable habitat for this species, and so there is a potential risk of CFP occurring when fish are caught and consumed from along the Kermadec arc. Toxicology studies will be carried out to determine whether CTX compounds that were not targeted in the analyses carried out in this study, or other non-related toxic compounds, are produced. 

Investigation into the genetic differences between toxic and non-toxic isolates of *G. polynesiensis* will be carried out and more strains of this species will be isolated and analysed for toxins in the future. The suite of species present at Macauley Island is similar to earlier isolations from Rangitahua, although *G. polynesiensis* was not successfully cultured from Rangitahua samples, but was detected by metabarcoding analysis (see paper by Smith et al., this issue of *Marine Drugs*) [35]. Both *G. honu* and *G. pacificus* were isolated from Rangitahua, bringing the number of species isolated from the Kermadec Islands to four (Figure 1). The type species, *G. toxicus*, was not found in the Kermadec Islands, but has been reported from New Caledonia [22], which is considered part of Zealandia. *Gambierdiscus* sp. has also been reported from mainland New Zealand [11]. One-third of the fifteen described *Gambierdiscus* species, twelve of which have been reported from the Pacific region [8], have therefore been isolated from the Kermadec/Zealandia region. All the species from the Kermadec Islands tested at Cawthron Institute have produced putative MTX-3, which has been termed ‘putative’ (reputed to be) due to the ambiguity surrounding the structure and its relation to other toxin analogues produced by *Gambierdiscus*. The parent mass is approximately one-third the MW of MTX. MTX-3 is ubiquitous to all *Gambierdiscus* species isolated from throughout the Pacific, although *G. carpenteri* isolates from NSW Australia have been shown not to produce it. Research is currently being conducted on the isolation and purification of MTX-3 with the aim of answering these fundamental questions.

The dominant co-occurring dinoflagellates were isolated during this study and could be used as indicators of potential *Gambierdiscus* presence for future sampling efforts, as they share the same habitat as *Gambierdiscus.* At both Macauley Island and Rangitahua [29,30] co-occurring dinoflagellate species included *Ostreopsis* sp. 3 and *Coolia malayensis*. The latter is a common dinoflagellate in mainland New Zealand’s northern waters [36], where *Fukuyoa paulensis* (previously *G.* cf. *yasumotoi*) has also been found [12,35]. The habitat is therefore suitable for *Gambierdiscus* to flourish, particularly with the warming seas recorded in Northland in recent years [8].

The risk of CFP occurring in New Zealand and its territories will continue to be assessed using traditional and molecular techniques, including high-throughput DNA sequencing. The risk is currently considered low in the Kermadec Islands, particularly as CTX is considered of greater concern for CFP than the MTXs produced by the commonly isolated *G. australes*. The potential for CFP incidents to occur in the future is, however, real as CTX-producing strains of *G. polynesiensis* occur throughout the Pacific and with more rigorous sampling it is only a matter of time before toxic strains are found at sites such as Rangitahua. As more *G. polynesiensis* isolates are tested for CTX production more data will be generated, and that data will be linked with the changing environmental conditions to inform predictive monitoring and so predict CFP. 

The risk of fish, particularly herbivorous fish, causing CFP in the Zealandia region is real, as *G. toxicus* (isolated from New Caledonia) [22] is considered toxic as determined by cell-based bioassays (although LC-MS/MS data is lacking). The risk for CFP occurring in mainland New Zealand is currently considered low, but with sea temperature increases and the expanding distribution of *Gambierdiscus* throughout the Pacific that risk will continue to be closely monitored.

## 4. Materials and Methods

### 4.1. Sampling, Isolation and Culture

Sampling was carried out on October 2016 by divers on the southeast coast of Macauley Island, Kermadec Islands (Lat. 30°23.75′ S, Long. 178°42.17′ W; Figure 1). Macroalgae attached to rocks were shaken into containers of local seawater to dislodge dinoflagellates. The macroalgae were then removed. Sub-samples were transferred to 50 ml tubes (Corning CentriStar, Shanghai, China) and germanium dioxide and f/2 medium [37] were added to suppress diatom growth and to encourage dinoflagellate growth respectively (1% final conc. each). Sampling was also carried out at L’Esperance Rock (Lat. 31°34.3 75′ S, Long. 17°83.03′ W).

Dinoflagellate cells were isolated as described previously [36] and transferred to 12-well tissue culture plates (Becton Dickinson, Franklin Lakes, NJ, USA) containing f/2 medium and filtered (0.35 µm), UV treated sea water. Culture conditions were: 25 °C (±2 °C); 40–70 µmol m^−2^ s^−1^ photon irradiance; 12:12 h L:D). Selected clonal cultures of the *Gambierdiscus* species isolated during this study are maintained in the CICCM.

### 4.2. DNA Sequencing and Phylogenetic Analyses of Dinoflagellate Cultures

Dinoflagellate cultures were centrifuged (542 *g*, 15 min, RT) and DNA extracted using a PowerSoil™ DNA isolation kit (Mo Bio Inc., Carlsbad, CA, USA). The D8-D10 region of the large subunit ribosomal RNA gene (LSU rDNA) was amplified as described previously [38] using primers D8F and D10R [39]. The PCR amplifications were carried out as described previously [38] and sequencing was carried out at Genetic Analysis Services, University of Otago (Dunedin, New Zealand). Sequences were aligned using the ClustalW algorithm [40] in Geneious^®^ v8.1.5 [41] and conflicts resolved by manual inspection. Sequences were aligned using the ClustalW algorithm in Geneious with publically available sequences from GenBank (www.ncbi.nlm.nih.gov). Bayesian analyses were carried out in Geneious^®^ using MrBayes 3.1.2 [42]. The evolutionary model (general time reversible with gamma-shaped among-site variation, GTR+G) was selected using MrModeltest v 2.2 [43]. The consensus sequences from all reads of each taxonomic assignment were aligned with references sequences. Analyses of alignments were carried out in two simultaneous runs with four chains each 2 × 10^6^ generations, sampling every 1000 trees, discarding a burn-in period of the first 1000 sampling points. After 2 × 10^6^ generations, potential scale reduction factor values were approximately 1.0 and average standard deviation of split frequencies were less than 0.01.

### 4.3. Toxin Analyses

*Gambierdiscus* cultures were centrifuged (3200× *g*, 15 °C, 15 min) and the growth media decanted to afford a cell pellet. Cell lysis was induced using a sonication bath (59 Hz, 10 min) and the toxins extracted twice with pure methanol (approx. 200,000 cells/mL). Each extract was analysed for selected algal CTXs analogues (LoD 1 ng/mL), MTX-1 (LoD 1 ng/mL) and a putative MTX analogue previously described as MTX-3 [44]. This was performed using a quantitative LC-MS/MS method developed at the Cawthron Institute (full method details will be disclosed in an upcoming manuscript). MTX-1 quantitation was performed using non-certified in-house reference material and therefore the results presented are for intra-study comparison only. LC-MS/MS analysis was carried out on a Waters Acquity UPLC i-Class system (Waters, Milford, MA, USA) coupled to a Waters Xevo TQ-S triple quadrupole mass spectrometer with electrospray ionization (Waters, Manchester, UK). Chromatographic separation used a BEH Phenyl column (Waters 1.7 μm, 100 × 2.1 mm column) and eluted with ammoniated mobile phases; (A) Milli-Q (0.2% NH4OH *v*/*v*) and (B) acetonitrile (0.2% NH_4_OH). Multiple reaction monitoring (MRM) transitions in ESI+, quantitative and qualitative, were established for the dominant [M + H]^+^ ions of the algal CTXs (CTX-3B; CTX-3C; CTX-4A; CTX-4B) and quantitation was performed using reference material provided by Dr. Mireille Chinain, Institut Louis Malardé, Tahiti, French Polynesia. MRM transitions to monitor the MTX-1 di-anion [M − 2H]^−^, in ESI-, were generated using material provided by Prof. Takeshi Yasumoto, Biochemistry and Food Technology Division, National Research Institute of Fisheries Science, Japan. Data acquisition and processing was performed with TargetLynx software (Waters-Micromass, Manchester, UK). Peak areas were integrated and sample concentrations calculated from linear calibration curves generated from calibration standards.

## 5. Conclusions

The genus *Gambierdiscus* occurs in New Zealand’s northeastern Kermadec Islands and throughout the Zealandia regions of the southwestern Pacific. Research will continue in these areas to ascertain the risk of ciguatera fish poisoning for New Zealand. The risk is currently extremely low, but is likely to increase with warming seas and the geographic expansion of *Gambierdiscus* to new areas.

## Figures and Tables

**Table 1:** Identification of *Gambierdiscus* isolates from Macauley Island, Kermadec Islands, by analysis of DNA sequencing data, and toxin production by the isolates as determined by LC-MS/MS.

Species	Isolate Code	CICCM Code	GenBank Accession Number	Toxins Produced (pg/cell) ^1^	
				Ciguatoxins ^2^	Maitotoxin-1
*G. polynesiensis*	Mac3-o	CAWD254	MF109032	ND	ND
*G. australes*	Mac1-b	CAWD255	MF109033	ND	36
*G. australes*	Mac2-a	n		ND	25
*G. australes*	Mac2-b	n		ND	12
*G. australes*	Mac2-c	n		ND	22
*G. australes*	Mac3-a	n		ND	14
*G. australes*	Mac3-b	n		ND	20
*G. australes*	Mac3-c	CAWD256	MF109034	ND	31
*G. australes*	Mac3-e	n		ND	19
*G. australes*	Mac3-i	n		ND	3
*G. australes*	Mac3-m	n		ND	19
*G. australes*	Mac3-n	n		ND	5
*G. australes*	Mac4-e	n		ND	10
*G. australes*	Mac4-f	n		ND	9
*G. australes*	Mac4-fuk	n		ND	21
*G. australes*	Mac4-g	n		ND	27
*G. australes*	Mac5-a	n		ND	16
*G. australes*	Mac5-b	n		ND	15
*G. australes*	Mac5-c	n		ND	8
*G. australes*	Mac5-e	n		ND	20
*G. australes*	Mac5-f	n		ND	8
*G. australes*	Mac5-g	n		ND	7
*G. australes*	Mac5-j	n		ND	32
*G. australes*	Mac5-l	n		ND	6
*G. australes*	Mac6-a	n		ND	18

^1^ All isolates tested produced MTX-3; ^2^ Ciguatoxins monitored were CTX-3B; CTX-3C; CTX-4A; CTX-4B; n: not deposited in the Cawthron Institute Culture Collection of Micro-algae (CICCM); ND: not detected.

## References

[1] Parsons M.L., Settlemier C.J., Ballauer J.M. (2011). An examination of the epiphytic nature of *Gambierdiscus toxicus*, a dinoflagellate involved in ciguatera fish poisoning. Harmful Algae.

[2] Parsons M., Aligizaki A., Bottein M.-Y.D., Fraga S., Morton S., Penna A., Rhodes L. (2012). *Gambierdiscus* and *Ostreopsis*: Reassessment of the state of knowledge of their taxonomy, geography, ecophysiology and toxicology. Harmful Algae.

[3] Selwood A., Rhodes L., Smith K., Harwood D.T., Mackenzie L. (2015). Development of two novel UPLC-MS/MS methods for the analysis of maitotoxin from micro-algal cultures. Marine and Fresh-Water Harmful Algae, Proceedings of the 16th International Conference on Harmful Algae, Wellington, New Zealand, 27–31 October 2014.

[4] Rongo T., van Woesik R. (2012). Socioeconomic consequences of ciguatera poisoning in Rarotonga, southern Cook Islands. Harmful Algae.

[5] Schep L.J., Slaughter R.J., Temple W.A., Beasley D.M.G. (2010). Ciguatera poisoning: An interesting occurrence in New Zealand. N. Z. Med. J..

[6] Palafox N.A., Jain L.G., Pinano A.Z., Gulik T.M., Williams R.K., Schatz I.J. (1988). Successful treatment of ciguatera fish poisoning with intravenous mannitol. J. Am. Med. Assoc..

[7] Schnorf H., Taurarii M., Cundy T. (2002). Ciguatera fish poisoning—A double-blind randomized trial of mannitol therapy. Neurology.

[8] Rhodes L., Smith K.F., Haywood T., Murray S., Biessy L., Argyle P., Munday R. (2017). Is *Gambierdiscus* expanding its geographic range in the Pacific region?. Harmful Algae News.

[9] Rhodes L., Smith K., Verma A., Curley B., Harwood T., Murray S., Kohli G.S., Solomona D., Rongo T., Munday R. (2017). A new species of *Gambierdiscus* (Dinophyceae) from the south-west Pacific: *Gambierdiscus honu*. Harmful Algae.

[10] Kohli G.S., Murray S.A., Neilan B.A., Rhodes L.L., Harwood T., Smith K., Mayer L., Capper A., Brett S., Hallegraeff G. (2014). High abundance of the potentially maitotoxic dinoflagellate *Gambierdiscus carpenteri* in temperate waters of New South Wales, Australia. Harmful Algae.

[11] Chang F.H. (1996). Shellfish toxin update. Seafood New Zealand.

[12] Rhodes L., Gimenez Papiol G., Smith K., Harwood T. (2013). *Gambierdiscus* cf*. yasumotoi* (Dinophyceae) isolated from New Zealand’s sub-tropical northern coastal waters. N. Z. J. Mar. Freshw. Res..

[13] Llewellyn L.E. (2010). Revisiting the association between sea surface temperature and the epidemiology of fish poisoning in the South Pacific: Reassessing the link between ciguatera and climate change. Toxicon.

[14] Kohli G.S., Farrell H., Murray S.A., Botana L.M., Louzao C., Vilariño N. (2015). *Gambierdiscus*, the cause of ciguatera fish poisoning: An increased human health threat influenced by climate change. Climate Change and Marine and Freshwater Toxins.

[15] Vergés A., Steinberg P.D., Hay M.E., Poore A.G.B., Campbell A.H., Ballesteros E., Heck K.L., Booth D.J., Coleman M.A., Feary D.A. (2014). The tropicalisation of temperate marine ecosystems: Climate-mediated changes in herbivory and community phase shifts. Proc. R. Soc. B.

[16] Bryan S.E., Cook A.G., Evans J.P., Hebden K., Hurrey L., Colls P., Jell J.S., Weatherley D., Firn J. (2012). Rapid, long-distance dispersal by pumice rafting. PLoS ONE.

[17] Wunderman R., Global Volcanism Program (2012). Report on Havre Seamount (New Zealand). Bulletin of the Global Volcanism Network.

[18] Mortimer N., Campbell H.J., Tulloch A.J., King P.R., Stagpoole V.M., Wood R.A., Rattenbury M.S., Sutherland R., Adams C.J., Collot J. (2017). Zealandia: Earth’s Hidden Continent. GSA Today.

[19] Baumann F., Bourrat M.-B., Pauillac S. (2010). Prevalence, symptoms and chronicity of ciguatera in New Caledonia: Results from an adult population survey conducted in Noumea during 2005. Toxicon.

[20] Chinain M., Faust M.A., Pauillac S. (1999). Morphology and molecular analyses of three species of *Gambierdiscus* (Dinophyceae): *G. pacificus*, sp. nov., *G. australes*, sp. nov., and *G. polynesiensis,* sp. nov.. J. Phycol..

[21] Litaker R.W., Vandersea M.W., Faust M.A., Kibler S.R., Chinain M., Holmes M.J., Holland W.C., Tester P.A. (2009). Taxonomy of *Gambierdiscus* including four new species, *Gambierdiscus caribaeus, Gambierdiscus carolinianus*, *Gambierdiscus carpenteri* and *Gambierdiscus ruetzleri* (Gonyaulacales, Dinophyceae). Phycologia.

[22] Laurent D., Kerbrat A.-S., Darius H.T., Girard E., Golubic S., Benoit E., Sauviat M.-P., Chinain M., Molgo J., Pauillac S. (2008). Are cyanobacteria involved in Ciguatera Fish Poisoning-like outbreaks in New Caledonia?. Harmful Algae.

[23] Roué M., Darius H.T., Picot S., Ung A., Viallon J., Gaertner-Mazouni N., Sibat M., Amzil Z., Chinain M. (2016). Evidence of the bioaccumulation of ciguatoxins in giant clams (*Tridacna maxima*) exposed to *Gambierdiscus* spp. cells. Harmful Algae.

[24] Marsden I.D., Contreras A.M., Mackenzie L., Munro M.H.G. (2015). A comparison of the physiological responses, behaviour and biotransformation of paralytic shellfish poisoning toxins in a surf-clam (*Paphies donacina*) and the green-lipped mussel (*Perna canaliculus*). Mar. Freshw. Res..

[25] Rhodes L., Smith K., Harwood T., Selwood A., Argyle P., Bedford C., Munday R., Mackenzie L. (2015). Gambierdiscus and *Ostreopsis* from New Zealand, the Kermadec Islands and the Cook Islands and the risk of ciguatera fish poisoning in New Zealand. Marine and Fresh-Water Harmful Algae, Proceedings of the 16th International Conference on Harmful Algae, Wellington, New Zealand, 27–31 October 2014.

[26] Gentry S. (2013). Raoul and the Kermadecs. New Zealand’s Northernmost Islands.

[27] Trnski T., de Lange P.J. (2015). Introduction to the Kermadec Biodiscovery Expedition 2011. Bull. Auckl. Mus..

[28] Duffy C.A.J., Ahyong S.T. (2015). Annotated checklist of the marine flora and fauna of the Kermadec Islands Marine Reserve and northern Kermadec Ridge, New Zealand. Bull. Auckl. Mus..

[29] Rhodes L., Smith K., Harwood T., Bedford C. (2014). Novel and toxin-producing epiphytic dinoflagellates isolated from sub-tropical Raoul Island, Kermadec Islands group. N. Z. J. Mar. Freshw. Res..

[30] Rhodes L.L., Smith K.F., Verma A., Murray S., Harwood D.T., Trnski T. (2017). The dinoflagellate genera *Gambierdiscus* and *Ostreopsis* from sub-tropical Raoul Island and North Meyer Island, Kermadec Islands. N. Z. J. Mar. Freshw. Res..

[31] Sato S., Nishimura T., Uehara K., Sakanari H., Tawong W., Hariganeya N., Smith K., Rhodes L., Yasumoto T., Taira Y. (2011). Phylogeography of *Ostreopsis* along west Pacific coast, with special reference to a novel clade from Japan. PLoS ONE.

[32] Mohammad-Noor N., Moestrup O., Lundholm N., Fraga S., Adam A., Holmes M.J., Saleh E. (2013). Autecology and phylogeny of *Coolia tropicalis* and *Coolia malayensis* (Dinophyceae), with emphasis on taxonomy of *C. tropicalis* based on light microscopy, scanning electron microscopy and LSU rDNA. J. Phycol..

[33] Pisapia F., Holland W.C., Hardison D.R., Litaker R.W., Fraga S., Nishimura T., Adachi M., Nguyen-Ngoc L., Séchet V., Amzil Z. (2017). Toxicity screening of 13 *Gambierdiscus* strains using neuro-2a and erythrocyte lysis bioassays. Harmful Algae.

[34] Munday R., Murray S., Rhodes L.L., Larsson M., Harwood D.T. (2017). Ciguatoxins and maitotoxins in extracts of 17 *Gambierdiscus* isolates from the South Pacific and their toxicity to mice by intraperitoneal and oral administration. Mar. Drugs.

[35] Smith K.F., Biessy L., Argyle P., Trnski T., Halafihi T., Rhodes L. (2017). Molecular identification of *Gambierdiscus* and *Fukuyoa* from environmental samples. Mar. Drugs.

[36] Rhodes L.L., Smith K.F., Gimenez Papiol G., Adamson J.E., Harwood T., Munday R. (2014). Epiphytic dinoflagellates in sub-tropical New Zealand, in particular the genus *Coolia* Meunier. Harmful Algae.

[37] Guillard R.R., Smith W.H., Chanley M.H. (1975). Culture of phytoplankton for feeding marine invertebrates. Culture of Marine Invertebrate Animals.

[38] Smith K.F., Rhodes L., Verma A., Curley B.G., Harwood D.T., Kohli G.S., Solomona D., Rongo T., Munday R., Munday S.A. (2016). A new *Gambierdiscus* species (Dinophyceae) from Rarotonga, Cook Islands: *Gambierdiscus cheloniae* sp. nov.. Harmful Algae.

[39] Litaker R.W., Vandersea M.W., Faust M.A., Kibler S.R., Nau A.W., Holland W.C., Chinain M., Holmes M.J., Tester P.A. (2010). Global distribution of ciguatera causing dinoflagellates in the genus *Gambierdiscus*. Toxicon.

[40] Thompson J.D., Higgins D.G., Gibson T.J. (1994). CLUSTAL W: Improving the sensitivity of progressive multiple sequence alignment through sequence weighting, position-specific gap penalties and weight matrix choice. Nucleic Acids Res..

[41] Kearse M., Moir R., Wilson A., Stones-Havas S., Cheung M., Sturrock S., Buxton S., Cooper A., Markowitz S., Duran C. (2012). Geneious Basic: An integrated and extendable desktop software platform for the organization and analysis of sequence data. Bioinformatics.

[42] Huelsenbeck J.P., Ronquist F. (2001). Mrbayes: Bayesian inference of phylogenetic trees. Bioinformatics.

[43] Nylander J.A.A. (2004). MrModeltest v2 Program Distributed by the Author.

[44] Holmes M.J., Lewis R.J. (1994). Purification and characterisation of large and small maitotoxins from cultured *Gambierdiscus toxicus*. Nat. Toxins.

